# Prospective study on magnetic resonance imaging in cochlear implant patients

**DOI:** 10.1007/s00405-023-08224-1

**Published:** 2023-09-14

**Authors:** Silke Helbig, Neele Thiemann, Elke Hattingen, Andreas Loth, Timo Stöver, Martin Leinung

**Affiliations:** 1 Department of Otorhinolaryngology, Head and Neck Surgery, University Hospital, Goethe University Frankfurt, Theodor-Stern-Kai 7, 60590 Frankfurt, Germany; 2 Department of Neuroradiology, University Hospital, Goethe University Frankfurt, Frankfurt, Germany

**Keywords:** Cochlear implant, Magnetic resonance imaging, Complications, Magnet dislocation, Head bandage

## Abstract

**Purpose:**

Monocentric, prospective study to investigate whether concomitant support of cochlear implant (CI) patients by CI-trained otolaryngologists and application of a standardized head bandage can minimize potential complications during magnetic resonance imaging (MRI).

**Methods:**

Thirty-seven patients with 46 CIs underwent MRI with a prophylactic head bandage. All participants and the otolaryngologist at the CI center completed pre- and post-MRI questionnaires documenting body region scanned, duration of MRI and bandage wear, field strength during the scan, and any complications. If pain was experienced, it was assessed using a visual analog scale (1–10).

**Results:**

MRI was performed without adverse events in 37.8% of cases. Magnet dislocation requiring surgical revision occurred in 2% of cases. Pain was reported in 86% of cases, often due to the tightness of the dressing. Patients with rotating, MRI-compatible magnets reported significantly less pain than participants with older-generation implants. In 11% of cases, the MRI was discontinued.

**Conclusion:**

Serious complications during MRI in cochlear implant patients are rare. Pain is the most common adverse event, probably mainly due to the tight bandage required by most implant types. With newer generations of magnets, these patients experience less pain, no dislocation of the magnets, and no need for bandaging. Although magnet dislocation cannot be completely prevented in older generations of implants, it appears to be reduced by good patient management, which recommends examination under the guidance of physicians trained in the use of hearing implants.

## Introduction and background

### Magnetic resonance imaging and its impact on hearing implants

Magnetic resonance imaging (MRI) has become increasingly important as a diagnostic tool in recent years because it provides better soft tissue contrast and, thus, greater diagnostic accuracy, and avoids potentially harmful radiation exposure. In 2018, for example, more than two million inpatient MRI scans were performed in Germany [1]. However, this examination also carries a potential risk for cochlear implant (CI) recipients that must be weighed against the diagnostic benefit.

Patients with cochlear implants may experience severe pain during MRI, which may even lead to discontinuation of the examination [2–4]. The lowest reported levels of pain and the highest rates of complete examination were found in studies that used local anesthetics to reduce pain during MRI [5, 6].

Displacement of the internal magnet caused by the magnetic field of the MRI is a serious risk, which in the past has led to a critical view or even contraindication of MRI in CI patients [7]. A prospective study by Tam et al. found a very low magnetic dislocation rate of 1.2% in 400 MRI at 1.5 T [5]. Fussell et al. reported on 79 patients and 157 hearing implants, respectively, in whom 131 MRIs were performed at 1.5 T and found 14 (9%) magnetic dislocations, half of which required surgical revision [6]. Other authors have also reported a higher risk of magnet dislocation, ranging from 3.1 to 9.1% [2–4, 8, 9]. However, these studies were mostly retrospective and the patient populations were smaller. In addition, the use of the head bandage recommended by the manufacturer was not reliably documented in all cases, and the magnetic field strength of 1.5 T recommended for CI patients was sometimes exceeded.

Induction of electric currents by rapidly changing gradient fields and emission of radiofrequency pulses could lead to local tissue heating with significantly higher absorption rates [10, 11]. However, studies with cochlear implants have not yet shown relevant temperature increases that would pose a risk to the surrounding tissue [12, 13]. Polarity reversal or demagnetization of the magnet during an MRI is a relatively rare complication that may result in reduced retention of the external speech processor [4, 8, 14, 15].

### Effect of head bandage during MRI

Gubbels and McMenomey demonstrated in a cadaveric model that a head bandage reduces the risk of magnet dislocation during MRI [16]. However, a head bandage does not appear to completely prevent magnet dislocation [17, 18]. In a retrospective study with an observation period of 13 years, Hassepass et al. found 23 cases of magnet dislocation in patients with CI. Ten of these cases occurred despite the use of a bandage without a counter-pressure element [19]. This suggests that either the bandage does not reliably reduce dislocation or the dressing technique needs to be improved. Finally, it does not seem to make a significant difference whether the preventive head bandage is applied by a trained radiologist or an otolaryngologist [6].

In fact, there are a number of different bandaging techniques to hold the magnet in place and each manufacturer offers its own bandaging material. Leinung et al. investigated the holding forces of different head bandages on a test model and showed that an elastic bandage with an additional cylindrical counter-pressure element was superior to other techniques [20].

It can, therefore, be concluded that the question of the safety of MRI for hearing implant recipients has not yet been conclusively resolved. For this reason, the present study will prospectively determine the adverse events reported by patients undergoing MRI when supervised by a CI-trained physician together with radiologists who are consistently aware of hearing implant conditions during the examination. In particular, our study will focus on the question of whether the consistent use of a standardized head bandage in this context makes the procedure safer.

## Method

### Compliance with ethical standards

Ethical approval was obtained from the local ethics committee (No. 390/17; 05/04/2018) before the start of the monocentric study, and a supplementary amendment was approved on 15/07/2020. There were no age restrictions, and all patients (or their parents) who were mentally and linguistically able to fulfill the study requirements were included. Informed consent was obtained from all participating patients prior to the study. All procedures within this study were in accordance with the ethical standards of the institutional and national research committee and with the Helsinki Declaration of 1964 and its subsequent amendments.

### Study population

**Fig. 1:**
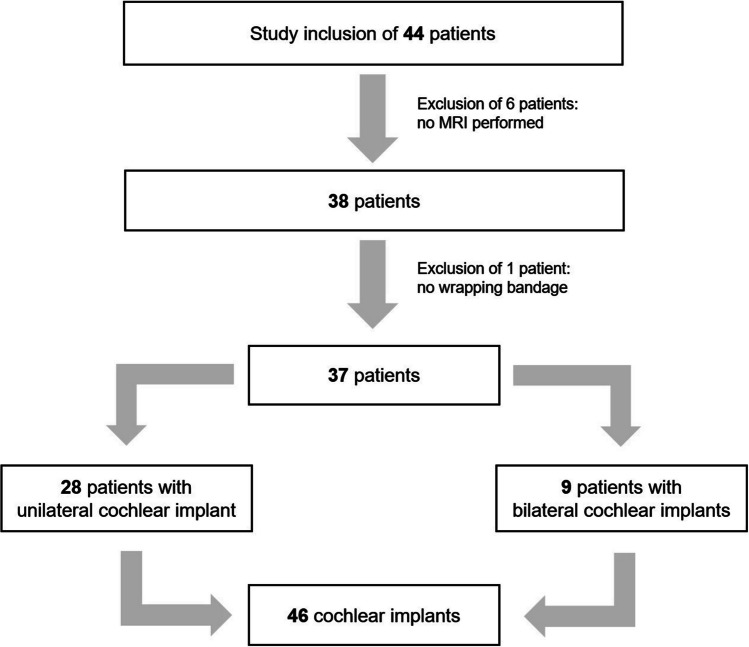
Overview of dropouts during the study. Thirty-seven patients (46 cochlear implants) were included in the final analysis

From May 2018 to December 2021, 44 patients were enrolled in this prospective study who were followed up after CI implantation in a university ENT clinic and who required MRI for various indications. During the course of the study, some patients had to be excluded from the final evaluation (Fig. 1). As a result, six patients did not receive the planned MRI. In two of these cases, the standardized bandage was too painful and was removed before the MRI, so the examination was not started. In one case, the patient refused the scan after being fully informed of the possible risks, and a CT scan was performed instead. Another patient had an X-ray instead of an MRI. In four other cases, imaging was refused by the treating radiologist, even though a head bandage had been applied in accordance with the manufacturer's instructions. Two of these patients had implants with magnets aligning itself during scanning and subsequently received the requested MRI in the neuroradiology department of a university hospital. One additional patient with a bilateral CI612 implant was subsequently excluded from the analysis because she had not received a head bandage in accordance with the manufacturer's instructions.

Thus, 37 patients (22 females, 15 males) were evaluated, of whom 28 had unilateral and nine had bilateral CIs, resulting in a total of 46 implants evaluated in 37 MRIs. The mean age was 57.68 ± 18.975 years, with the youngest patient being two years old and the oldest being 86 years old. Pre- and post-MRI questionnaires were obtained from all 37 patients (46 implants) included in this study.

Progressive sensorineural hearing loss was the most common reason for cochlear implantation in 15 patients. This was followed by sudden hearing loss in 11 patients. Implants from three manufacturers were represented in the study population, with implants from *Cochlear* and *MED-EL* being the most common. Overall, 13 of the 46 implants were of the newer generation with MRI-compatible magnets that align in the magnetic field. Table 1 shows a detailed breakdown of the individual implant type and etiology for all patients.

**Table 1:** Etiology of deafness, age, sex, and implant-type data for the 37 patients included in the study

Patient		Etiology	Cochlear implant	Cochlear implant
Sex	Age		Right side	Left side
f	83	Sudden hearing loss	Cochlear CI422	–
m	51	Progressive sensorineural hearing loss	Cochlear CI512	Cochlear CI532
**f**	46	Iatrogenic auditory nerve injury	–	Cochlear CI532
f	86	Progressive sensorineural hearing loss	MED-EL Concerto	–
f	57	Sudden hearing loss	–	MED-EL Sonata
m	70	Sudden hearing loss	–	MED-EL Synchrony
m	2	Meningitis	MED-EL Synchrony	MED-EL Synchrony
f	60	Progressive sensorineural hearing loss	–	MED-EL Concerto
m	42	Ototoxic (otitis media)	–	MED-EL Synchrony
m	56	Sudden hearing loss	Cochlear CI24RE	Cochlear CI512
f	60	Progressive sensorineural hearing loss	Cochlear CI512	Cochlear CI512
f	69	Progressive sensorineural hearing loss	MED-EL Synchrony	–
m	62	Progressive sensorineural hearing loss	MED-EL Concerto	MED-EL Concerto
m	74	Progressive sensorineural hearing loss	–	Advanced Bionics HiRes 90 K
m	51	Sudden hearing loss	Cochlear CI512	–
m	51	Sudden hearing loss	–	MED-EL Concerto
m	76	Superficial siderosis	MED-EL-Synchrony	MED-EL-Synchrony
f	77	Progressive sensorineural hearing loss	–	Cochlear CI512
f	47	sudden hearing loss	Cochlear CI512	–
f	15	Unknown	MED-EL Pulsar CI100	–
f	53	Congenital	Cochlear CI24RE	MED-EL Concerto
f	78	Progressive sensorineural hearing loss		MED-EL Concerto
f	46	Labyrinthitis	Cochlear CI512	–
f	68	Sudden hearing loss	MED-EL Synchrony	–
m	55	Congenital	Cochlear CI24RE	–
f	56	Progressive sensorineural hearing loss	Cochlear CI24RE	–
f	50	Sudden hearing loss	Cochlear CI512	–
f	76	Progressive sensorineural hearing loss	MED-EL Synchrony	–
f	69	Progressive sensorineural hearing loss	MED-EL Synchrony 2	–
f	50	Congenital	–	Cochlear CI24RE
m	82	Sudden hearing loss	–	Cochlear CI512
m	67	Traumatic after accident	–	MED-EL Synchrony
f	28	Progressive sensorineural hearing loss	MED-EL Pulsar	MED-EL Pulsar
f	19	Progressive sensorineural hearing loss	Cochlear CI422	Cochlear CI512
m	62	Sudden hearing loss	MED-EL Synchrony	–
m	74	Intracochlear schwannoma	–	Cochlear CI612
f	66	Progressive sensorineural hearing loss	Advanced Bionics HiRes Ultra	–

Cochlear implants from three manufacturers were included in this study: *MED-EL* Innsbruck, Austria/ *Cochlear* Sydney, Australia/ *Advanced Bionics* (Sonova Holding AG), Los Angeles, California, USA

### Questionnaires

Participants were asked to attend the ENT outpatient clinic twice: once before and once after the MRI scan. Before the radiological examination, each patient was first assessed to determine whether there was a reasonable indication for MRI. Patients were then given detailed information about possible risks, given detailed information about the study procedure, and asked to sign the informed consent form. Patients were then asked to complete a questionnaire (Table 2), which included the type of implant and the date and location of the planned MRI scan. Patients were also asked to indicate which part of the body was to be imaged. The patient was then examined to ensure that there was no pathology in the area of the implant prior to the MRI. A further questionnaire documenting the patient's implant type and the type of head bandage used was completed by the physician applying the bandage. Any shaving over the magnet and any immediate bandage slippage was also documented (Table 3). All patients received a head bandage, regardless of implant type and even if this was not required according to the manufacturer's instructions (twelve Synchrony implants (*MED-EL, Innsbruck, Austria)* and one CI612, *Cochlear, Sydney, Australia*)).

**Table 2:** Questionnaires for the patients, in 1b. question 5a was added after the amendment

**a**	**Pre MRI**	
1	Which hearing implant (manufacturer, type) do you have (right/left/both sides)?	
2	What should be examined in the MRI?	
3	When should the MRI be performed?	
4	In which clinic, department or private practice should the MRI examination be performed?	
**b**	**Post MRI**	
1	Were you also in the closed MRI tube with your head during the MRI exam?	Yes/No
2	How long (in minutes) did the examination take?	
3	Was a wrapping bandage worn during the examination?	Yes/No
4	How long did you wear the wrapping bandage on the day of the examination	Less than 1 h 1 h to 2 h > 2 h to 3 h > 3 h to 4 h > 4 h to 5 h > 5 h
5	Did you have any pain in the implant area during the examination?	Yes/No
5a	*Amendment:* In case you experienced pain, what was the reason for it?	The wrapping bandage Pain arose during the MRI Both, wrapping bandage and MRI procedure
6	Please indicate on a scale of 0–10 how severe the pain was (0 = no pain 10 = pain as severe as can only be imagined)	
7	Did any other accompanying symptoms occur? If yes, what were they (multiple answers possible)?a	None Hearing loss Tinnitus Nausea/vomiting Anxiety Sweating Other
8	Did you discontinue the MRI exam?	Yes/No
9	If you discontinued the MRI exam, why did you discontinue the exam?	Claustrophobia Pain Other reason
10	Did you have any swelling on the implant immediately after the examination?	Yes/No
11	After the examination, did you have the impression that the speech processor of your implant does not hold as well since the MRI examination?	Yes/No
12	Did you have the impression after the examination that you hear worse on the implant ear?	Yes/No

**Table 3:** Physician questionnaires

**Pre MRI**		
1	Which implant is provided on the right/left side?	Cochlear implant Vibrant Soundbridge Bonebridge BAHA Attract CoDACS None
2	Was the patient shaved over the magnet prior to wrapping?	Yes/No
3	Was the patient shaved over the magnet prior to wrapping?	Yes/No
4	What wrapping bandage was applied?	None Wrap Thin counter-pressure element + wrap Cylindrical counter-pressure element + wrap
5	Did slippage of the wrap already occur during wrapping?	Yes/No
**Post MRI**		
1	Which body region was scanned?	
2	With which magnetic field strength (Tesla) was the examination performed?	
3	Are complications in the CI area recognizable (pressure point/ open wound/ swelling/ magnet palpable through skin)?	Yes/No
4	Was a radiological check of the implant performed after the MRI (X-ray, DVT, CT)?	Yes/No
5	If a radiological check of the implant was performed, how are the findings to be assessed?	No dislocation of the magnet Dislocation of the magnet Dislocation cannot be clearly assessed
6	Was immediate manual repositioning performed?	Yes/No
7	Is a surgical revision planned?	Yes/No
8	What are the current findings after MRI examination (multiple answers possible)?	MRI performed No complaints Pain in the implant area Swelling in the implant area Dislocation of the magnet, manual repositioning Dislocation of the magnet, surgically repositioned MRI aborted No complaints Pain in the implant area Swelling in the implant area Dislocation of the magnet, manual repositioning Dislocation of the magnet, surgically repositioned

The MRI was then performed either by the radiology department of the university hospital itself or by external radiology practices. Patients were allowed to remove the head bandage immediately after the scan and were seen in the ENT clinic immediately after the MRI or on one of the following days to record any complications. The implant site was re-examined for pressure points, open wounds, swelling, magnet dislocation or pain. The clinician documented this in a questionnaire (Table 3). Consequences of suspected magnet dislocation were also recorded, such as X-ray, manual or surgical repositioning of the magnet. The field strength at which the MRI was performed was also recorded. Patients were then asked to complete an additional questionnaire (Table 2). This recorded the duration of the head bandage and the duration of the MRI. Complications during and after imaging were asked about: tinnitus, hearing loss, nausea/vomiting, sweating, anxiety and pain were asked about using a visual analog scale of 1–10. They were also asked whether the MRI had been stopped. In cases where patients reported complications, they were contacted again to find out whether these were temporary or permanent. The data collected from the questionnaires were reviewed and compared with the requested radiological examination data and the electronic patient record.

### Head bandage

The head wrapping bandage that was used in this study followed a basic, standardized procedure. First, the implant magnet was located and marked on the scalp skin. If there was a lot of hair, a small area was shaved directly over the magnet and a non-compressible counter-pressure element was attached directly over the magnet with double-sided adhesive tape. During the course of this study, the manufacturers made changes to the material of the compression bandage and to the counter-pressure element. As soon as these became available, the officially approved counter-pressure elements, some of which were held in place magnetically (in which case no adhesive was needed), were used and fixed in the same position. In the case of bilateral hearing implants, this procedure was performed on both sides. An elastic bandage was then tightly wrapped around the head and fixed in place with adhesive strips in the forehead and lateral neck area to prevent it from slipping back. Finally, the counter-pressure element was checked to ensure that it had not slipped when the bandage was applied. Two patients with Synchrony implants refused the additional counter-pressure element and in these cases, compresses were placed over the magnet and the bandage was wrapped.

### Statistical analysis

The results of this study were analyzed using SPSS version 27 (IBM Corp.). Graphs and charts were then generated using either SPSS or Excel version 2013 (Microsoft Corp.). Correlations between metric and ordinal scaled data were calculated using Spearman's correlation coefficient. A correlation coefficient of 0–0.25 was defined as a weak correlation, between 0.25 and 0.5 as a moderate correlation, 0.5–0.75 as a strong correlation, and above 0.75 as a very strong correlation. The Wilcoxon–Mann–Whitney *U* test was used for two independent samples and the Kruskal–Wallis test for more than two independent samples. The secondary outcome measures were analyzed descriptively and presented graphically using box plots, histograms, bar charts and pie charts.

## Results

### MRI indication and procedure

**Fig. 2:**
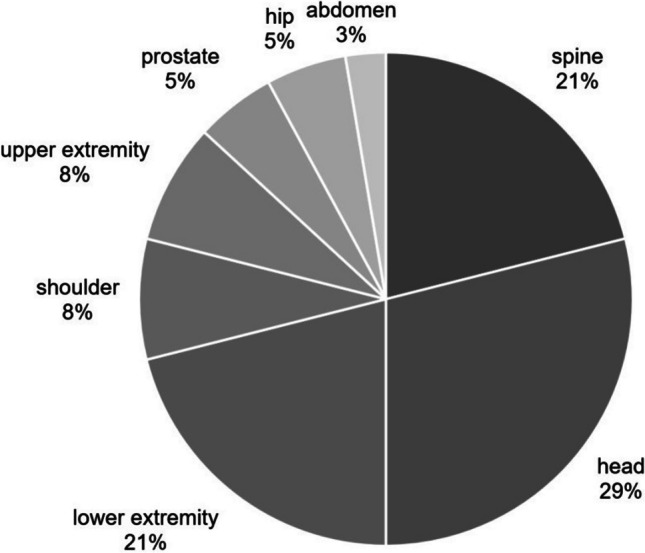
Overview of body regions scanned in all study patients. Examinations of the head and the spinal column accounted for approximately half of the examinations

The indications for the 37 MRI scans varied widely. In one patient, both the head and the spine were imaged in a single MRI scan, resulting in a total of 38 regions of the body being scanned. In particular, examinations of the head (*n* = 11) and spine (*n* = 8) were common, accounting for about half of the examinations. This was followed by examinations of the lower extremities (*n* = 8). Figure 2 provides an overview of the body regions scanned in all study patients.

The majority of MRIs, namely 2/3 (25/37), were not performed in a university institution but in radiology practices or radiology departments of external hospitals. The field strengths used were 1.5 T in 33 examinations. Only 4 cases were exceptions: one skull scan of a patient with a unilateral Synchrony CI was performed at 3 T, and three others were performed at 1 T. The duration of the MRI was reported by 35 of the 37 patients and averaged 23 min, with a minimum of 5 min and a maximum of 150 min. This longer examination time was explained by the examination of the head, cervical, thoracic and lumbar spine in one MR session. Thirty-five/37 patients responded to the question of whether the head was positioned inside or outside the MR scanner during the examination. In 65% of cases (24/35), the head was inside the MR gantry. The wrapping bandage was worn for an average of 152 min, with a maximum of 24 h and a minimum of less than one hour.

### Magnetic resonance imaging complications

Overall, 37.8% (14/37) of MRIs with the head bandage were performed without complications. In all other cases, either the patient or the clinician reported one or more complications in the questionnaire. These included accompanying vegetative symptoms, which are not specific to CI patients but can occur during this examination in general, such as anxiety, excessive sweating, nausea, vomiting during the examination.

### Displacement of the magnet

**Fig. 3:**
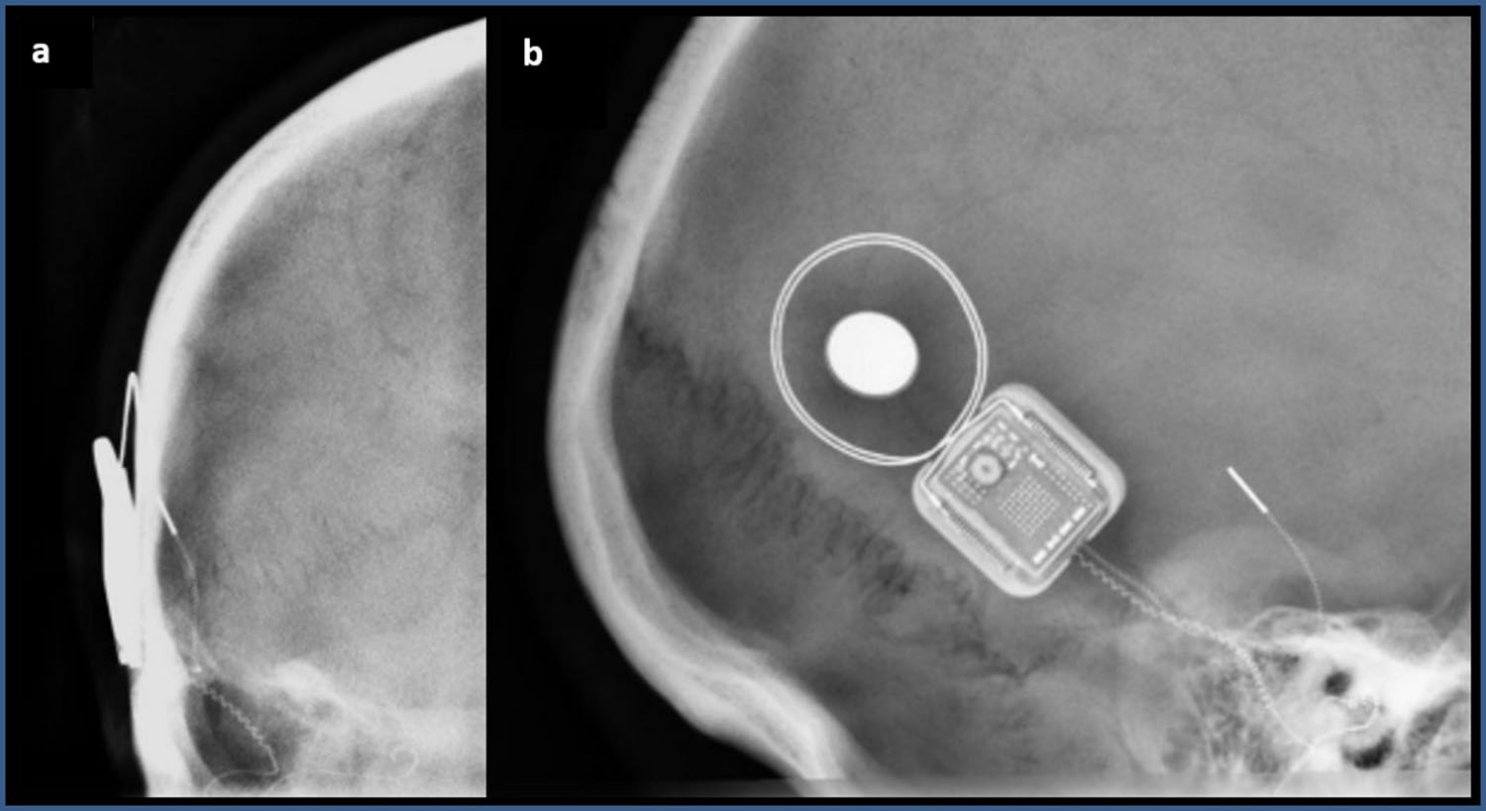
Radiographs of suspected and later confirmed magnet dislocation. **a** Radiograph of the skull anterior–posterior to show the case tangentially. The tilt of the magnet can be seen in the image. **b** Skull X-ray laterally, looking at the case from above. An off-center position of the magnet in the coil area can be seen

Since the risk of dislocation of the internal magnet is twice as high in bilaterally implanted patients, the following evaluation refers to implanted ear by MRI. Within the scope of 37 MRIs with applied head bandages, 46 implants could be included in the evaluation. The overall rate of magnet dislocation in this study was 2.17% (1/46). If new generations of implants with self-aligning magnets (Synchrony and CI612) are excluded, as there is a high probability that no dislocation will occur, the rate increases to 3% (1/33). Displacement of the internal magnet occurred in one patient with a unilateral CI512. The head MR scan was scheduled at 1.5 T but had to be stopped after five minutes due to severe pain with a visual analog scale (VAS) score of 10 out of 10. A direct medical examination followed: a dislocated magnet could be felt under the skin above the coil. However, the patient initially refused imaging to confirm the diagnosis. Manual pressure was applied to the magnet and the swelling reduced. Five months later, the patient presented for a scheduled routine check-up, and again painful swelling around the coil was confirmed, which had already caused the patient's use of the CI to be severely restricted. This time the patient consented to imaging, which revealed a dislocation of the internal magnet (Fig. 3). Despite the offer of immediate revision, she agreed to revision one month later. The implant was preserved and wound healing was uneventful.

In another case, a 1.5-T MRI scan of a bilaterally implanted patient was stopped due to pain on the right side where a CI422 implant was implanted. Swelling and tenderness were also noted after primary palpation. Radiological follow-up showed no evidence of dislocation.

### Pain perception

The study population also included a two-year-old child with bilateral Synchrony implants. As the examination was performed under general anesthesia, this child was excluded from the pain perception assessment. Therefore, the following evaluations refer to 36 MRIs performed with the head bandage in place.

**Fig. 4:**
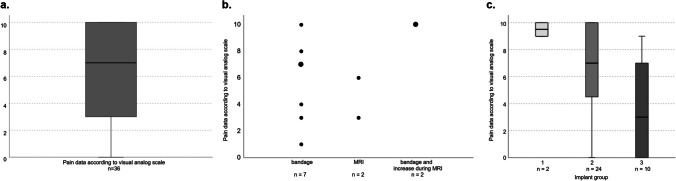
**a** Boxplot for the results of reported pain in the total study group (*n* = 36). **b** Scatter plot showing causes of pain during examination and individual pain data (mean 7, interquartile 5) in the 'amendment' group (*n* = 11). Tightness of the dressing was reported as the main cause of pain. **c** Boxplot for the results of reported pain in the three categorized implant housing groups. *1* implants with ceramic housings and magnets that do not align their poles in the MRI; *2* implants with silicone-titanium housings and magnets that do not align their poles in the MRI; *3* patients with silicone-titanium housings that align in the magnetic field during the MRI. Pain was significantly less in the third group

The mean VAS score for reported pain during the MR examination was seven, with a minimum of 0 and a maximum of 10. The interquartile range was seven (Fig. 4a). In five out of 36 cases (14%) the MRI was painless. In all other cases (86%), patients reported pain during the scan. In two cases, patients were even given oral painkillers during the preliminary interview because of the painful wrapping bandage. When it was realized that the bandage itself was causing significant pain, the questionnaire was extended. In an amendment dated 15/07/20, the question was added as to whether the pain could be explained by the head bandage, whether it occurred during the MRI, or whether both the head bandage and the MRI procedure caused pain. Eleven patients responded, and the following graph gives an overview of the causes of pain. In the majority of cases (7/11) the pain was caused by the tight bandage. The mean score for reported pain within this "amendment" group was also seven, with a minimum of one and a maximum of 10. The interquartile range was 5.0 (Fig. 4b).

### Correlation of pain with other factors

A total of eleven unilaterally or bilaterally implanted study participants had the newer generation of implants with rotating magnets suitable for MRI (12 Synchrony, 1 CI612), including the child who was scanned under general anesthesia. Therefore, ten implants with freely rotating magnets were included in the analysis. This group reported pain-free scans in three cases and pain in seven cases. In six of these cases, the patients stated, either in free text as a comment or in the modified questionnaire, that the pain was due to the intense pressure of the bandage. In one case, this even led to the MRI having to be stopped. In another case with a unilateral Synchrony implant, it was stated that the pain (VAS: 3/10) was caused by the MR scan alone.

Three groups were created to investigate the occurrence of pain according to the type of implant. The first two groups consisted of patients with non-rotating magnets, with implant group 1 consisting of a ceramic housing and group 2 consisting of a silicone-titanium housing. The third group was implanted with the newer generation of magnets (Synchrony, CI612). The Kruskal–Wallis test showed a significant difference in pain perception between the implant groups (*p* = 0.019). The subsequent Bonferroni-corrected post hoc test showed a significant difference between group 2 and group 3 (*p* = 0.043). The results for these three groups are shown in Fig. 4c. Patients with newer implant types and freely rotating magnets had significantly less pain during the study.

To identify other possible associations with pain during MRI, Spearman correlations were also examined for the following factors: duration of MRI, duration of bandage wear, and field strength level. No significant correlation was found for any of these factors. The Wilcoxon–Mann–Whitney *U* test was used to determine whether patients whose head was inside the MR gantry during the scan tended to experience more pain than patients whose head was outside the gantry. There was no significant difference between the two groups (*p* = 0.985).

### Discontinued examinations

In total, 10.8% (4/37) of the MRIs were interrupted because of pain. Only one scan could not be completed initially because of claustrophobia, but was then resumed and completed.

### Other complications

None of the patients experienced a permanent reduction in the magnetic holding force of their processor. Three patients (2 bilateral, 1 unilateral implanted) experienced a temporary decrease in magnetic retention of the speech processor after 1.5-T MRI, most likely due to swelling. No demagnetization occurred in the study population. Furthermore, there were no cases of damage to the electronics of the cochlear implant or complete device failure.

Three patients reported tinnitus, but in all cases the tinnitus was present before the MRI scan and was not increased or affected by the procedure. Seven patients in this study experienced temporary erythema or pressure points.

## Discussion

In this study, 37 patients were examined and complications were recorded in a timely and differentiated manner using questionnaires and ENT examinations. The aim was to reduce potential complications during MRI by providing optimal care for the CI patients. This included the use of a standardized head bandage and information from the CI-trained ENT physician to all those involved in the procedure.

To date, due to the exclusive study population (CI and MRI), there are few prospective studies with larger patient numbers on the MRI procedure in hearing implant recipients, and when available, these studies have several limitations. Tam et al. prospectively studied the largest group to date of 97 patients with brainstem or cochlear implants who underwent 400 MRIs, although pain and complications were not queried in detail and the type of preventive head bandage was not explained [5]. In the available retrospective studies, the use of a head bandage was not reliably documented in all patients and the method of implementation varied [3, 19]. In the study by Kim et al. 2 of the 18 patients studied did not receive a head bandage and were also scanned at 3 T, which is contrary to the manufacturer's recommendations for the hearing implants used [4]. There is evidence that this increases the rate of complications. For example, in the study by Shew et al. one case of dislocation occurred in the absence of a documented preventive bandage [2]. Walton et al. reviewed 76 MRIs in 13 patients with neurofibromatosis II, most of whom had brainstem implants. Again, dislocation occurred in one case where a head bandage with the usual counter-pressure element had not been applied, requiring revision surgery [21]. It can be concluded that these retrospective studies also highlight the general shortcomings that currently exist in the MRI procedure in CI, which again emphasizes the importance of these patients being cared for by hearing implant specialists. For this reason, suggestions to optimize this procedure, as presented here, are important to make it safer for this group of patients.

### Indication and conduct of magnetic resonance imaging

The indication for the scan is usually made and justified by the referring physician. The patient then contacts the radiologist directly or as within this study, the cochlear implant center. The cochlear implant specialists in this study then initiated the indication check through the referring external colleague and also informed the patients about the potential risks of this procedure. The radiologist reviewed the indication and determined whether the question could be answered by another, safer method or modality. If not, it is the radiologist’s responsibility to ensure that the patient is fully informed of the potential side effects and complications. The head bandage has been applied by the cochlear implant specialist as part of the trial, but the radiologist is responsible for its correct application and must check the head bandage. The patient or health insurance company has no claim for compensation if an incident occurs with correct indication, information, adherence to the time interval between information and examination and correct head bandage.

In all cases presented here, the magnetic field strength was chosen according to the manufacturer’s specifications. Predominantly 1.5 T was used, which is comparable to other studies [2, 3, 5, 6, 8, 22, 23]. Eleven patients underwent imaging of the head. As the head is the most frequently imaged region in inpatient MRI in all patient groups [1], it is not surprising that this part of the body has also been predominantly scanned in many other studies of hearing implant patients [4–6, 8, 22, 23].

### Complications during magnetic resonance imaging

Overall, magnet dislocation, a serious complication, occurred in one in 37 MRIs, or 2% of the cases in this study. Thus, the risk of magnet dislocation with MRI can be considered very low compared to the literature, which reports significantly higher rates of up to 9.1% of magnet dislocation [2–4, 8, 9]. This may be because previous study populations included older implant models with non-rotatable magnets. If, for this reason, the new generation of self-aligning magnets is excluded from the assessment in this study, the risk increases to 3%, which is still low. However, to the authors’ knowledge, this is the first prospective study in which a prophylactic head bandage applied by an otolaryngologist was consistently used and a fixed doctor’s visit was scheduled immediately before and after the MRI examination. Thus, this could explain the reduced dislocation rate. The implant model is an obvious influencing factor in this complication. A prospective study by Tam et al. also found a low magnetic dislocation rate of 1.2% [5]. The implants in question had a magnet housing of the same design as the implant in which the complication occurred in this study. In fact, the literature confirms a higher risk of magnet dislocation for the CI 24 and CI 5 series models [2, 4, 5, 8, 23]. An experimental model confirmed the increased risk for the CI24, but also for the Advanced Bionics HighRes Ultra, depending on the head orientation [24]. In addition, other studies and case reports report dislocations of this implant model [5, 28, 30]. In contrast, dislocation did not occur in any implant model with aligning magnets in this study, an observation also reported by other authors [2, 6].

In this study, most of the MRIs were performed in external radiology departments, a fact that is consistent with the literature, where it was also found that imaging was performed in most cases without the knowledge of the implanting clinic [9]. This is due to the design of the German healthcare system, where clinics are usually not allowed to perform outpatient examinations. The involvement of different practices makes it difficult to implement standardized working procedures. In such a setting, it seems more important to consult all participants before the examination and to control complications after the MRI. The setting described in this study, as well as the application of the head bandage by an implant-experienced physician, is reasonable and addresses this problem. It showed lower rates of internal magnet dislocation than those reported in large retrospective studies. For example, Loth et al. reported a dislocation rate of 7% in their retrospective study of 711 patients [9], compared to 2% in this study. This suggests a lower risk of magnet dislocation if the procedure is adequately supervised. However, since the risk of dislocation cannot be completely avoided in the older implant models described, even with a head bandage, it would be necessary to include a note on this in the information provided to this group of patients. Conversely, it would make sense to inform the radiologists who have so far generally rejected MRI in CI patients because of the risk of magnet dislocation. The knowledge that new implants are excluded from this could lead to a rethink.

The mean pain score during MRI in this study was seven (median) on the VAS. Four patients who reported pain of 9/10 or more on the VAS decided not to have the MRI scan. However, there were also patients who reported no pain at all. This wide variation in pain reporting is consistent with other studies. Pross et al. described pain in 27 patients, with a mean pain score of 4.6/10 on the VAS [22], and other authors also reported lower pain rates. However, in many of these studies, the administration of local anesthetics around the implant or the administration of systemic benzodiazepines influenced the results [5, 6, 15, 23]. In general, however, scatter in pain reports is not an uncommon phenomenon, even in the same patient undergoing multiple examinations [4]. In particular, pediatric patients received MRI under general anesthesia or at least analgesia in most studies [4, 8, 25, 26], and in this study, an infant was also examined under general anesthesia.

Other studies and case reports describe severe pain often associated with magnet dislocation that has occurred [4, 8, 27, 28]. Again, in this study, one patient with confirmed magnet dislocation reported a VAS score of 10/10, but with twelve other patients reporting scores of 9 or higher, it must be concluded that pain is not a reliable indicator of this complication. Rather, the main cause of pain in this study was the tightness of the bandage. As the latest implant modifications with rotating magnets no longer require a bandage during the examination (according to the manufacturer's guidelines), an improvement for patients in this respect can be expected in the future.

Furthermore, in our study there was no correlation between pain and the duration of the MRI examination or the fact that the head was positioned in the isocenter of the MR gantry. These findings are consistent with the literature [22]. Our data show that 10.8% of MR examinations were discontinued because of pain. In comparison, the literature shows not only lower discontinuation rates of 2.25% [5] and 6.1% [3], but also higher values such as in the retrospective study by Kim et al. in which five out of 30 cases (17%) were discontinued [4]. In general, other authors also report pain-related discontinuation [2, 6, 15, 22].

There was no evidence of demagnetization in this study. Three patients reported that the speech processor did not fit as tightly after the study as before. This could be an indication of demagnetization or simply a consequence of swelling after a tight head wrap. As the magnetic hold was only temporarily reduced in these cases and no stronger external magnet was used, demagnetization is unlikely. In addition, there was no polarity reversal of the magnet in any of the cases, which has also been reported in the literature [8, 15].

In addition, there were no cases in which the electronics of the cochlear implant were damaged or there was a complete failure of the device. This is consistent with other studies [2, 5, 8, 15, 19, 23] and supports the claim that current generations of cochlear implants are MRI compatible.

Three patients in the study reported tinnitus as a complication following MRI. Noise perception during MRI has rarely been described in cochlear implant recipients. For example, Shew et al. reported a patient with a cochlear implant CI24RE who had to discontinue a 1.5-T MRI because of pain and noise perception [2], and Holtmann et al. also reported the occurrence of noise perception in an implant patient [29].

Temporary erythema or pressure sores occurred in seven patients, most likely due to the head bandage worn, and were also described by Pross et al. who reported temporary skin redness in 29% of cases [22].

### Limitations

With 37 participants, the study group was relatively small and prospective studies with larger numbers of cases would be desirable to investigate the occurrence of complications and other influencing variables. Due to the low complication rate in this study, further analysis of factors leading to magnet dislocation or other complications was not possible. Therefore, possible associations between individual implant models and an increased likelihood of certain complications, such as magnet dislocation, could not be determined here. With a larger number of cases, a multivariable logistic regression model could be used to further investigate factors influencing the occurrence of individual complications.

## Conclusion

Today, CIs enable a growing number of patients to manage their hearing loss. However, a problem arises when these patients require MRI, as this type of imaging cannot be performed without risk with many of the CIs currently in use. In this study, 37.8% of patients underwent MRI under the supervision of an experienced clinician using a head wrap, with no adverse effects.

Pain was the most common adverse effect of MRI in CI patients and was responsible for almost all discontinuations. It was striking that patients with newer generations of implants with self-aligning magnets reported significantly less pain.

These implants also have advantages in terms of the risk of magnet dislocation. While magnet dislocation could not be completely prevented in the group of older implant models (3% of cases) even under the optimized conditions described in this study, no dislocations occurred with the new implants with self-aligning magnets.

Therefore, after an individual risk assessment, it seems reasonable to inform all parties involved about the risks of the different implants. While newer generations of implants could be subjected to external MRI without any problems if the manufacturer's conditions were observed, there is a need for clarification in the case of older generations of implants.

To ensure that implants at risk can also be subjected to MRI examination, it is necessary to provide expert care for CI patients during the imaging procedure. This could be regulated by standard operating procedures, of which the procedure outlined here could be a variant. These would help to ensure compliance with the manufacturer's precautions and the MR parameters required for each type of implant.

Further studies would be useful to consolidate the MRI safety conclusions for currently implanted CIs and to increase the safety of imaging older models. This may help to address the current refusal of CI patients by radiological centers.

## Data Availability

Not applicable
